# Acute Knockdown of Uncoupling Protein-2 Increases Uncoupling via the Adenine Nucleotide Transporter and Decreases Oxidative Stress in Diabetic Kidneys

**DOI:** 10.1371/journal.pone.0039635

**Published:** 2012-07-02

**Authors:** Malou Friederich-Persson, Shakil Aslam, Lina Nordquist, William J. Welch, Christopher S. Wilcox, Fredrik Palm

**Affiliations:** 1 Division of Integrative Physiology, Department of Medical Cell Biology, Uppsala University, Uppsala, Sweden; 2 Division of Nephrology and Hypertension, Department of Medicine, Kidney and Vascular Research Centre, Georgetown University Medical Center, Washington, D.C., United States of America; 3 Department of Medical Health Sciences, Linkoping University, Linkoping, Sweden; Medical University of South Carolina, United States of America

## Abstract

Increased O_2_ metabolism resulting in chronic hypoxia is common in models of endstage renal disease. Mitochondrial uncoupling increases O_2_ consumption but the ensuing reduction in mitochondrial membrane potential may limit excessive oxidative stress. The present study addressed the hypothesis that mitochondrial uncoupling regulates mitochondria function and oxidative stress in the diabetic kidney. Isolated mitochondria from kidney cortex of control and streptozotocin-induced diabetic rats were studied before and after siRNA knockdown of uncoupling protein-2 (UCP-2). Diabetes resulted in increased UCP-2 protein expression and UCP-2-mediated uncoupling, but normal mitochondria membrane potential. This uncoupling was inhibited by GDP, which also increased the membrane potential. siRNA reduced UCP-2 protein expression in controls and diabetics (−30–50%), but paradoxically further increased uncoupling and markedly reduced the membrane potential. This siRNA mediated uncoupling was unaffected by GDP but was blocked by ADP and carboxyatractylate (CAT). Mitochondria membrane potential after UCP-2 siRNA was unaffected by GDP but increased by CAT. This demonstrated that further increased mitochondria uncoupling after siRNA towards UCP-2 is mediated through the adenine nucleotide transporter (ANT). The increased oxidative stress in the diabetic kidney, manifested as increased thiobarbituric acids, was reduced by knocking down UCP-2 whereas whole-body oxidative stress, manifested as increased circulating malondialdehyde, remained unaffected. All parameters investigated were unaffected by scrambled siRNA. In conclusion, mitochondrial uncoupling via UCP-2 regulates mitochondria membrane potential in diabetes. However, blockade of the diabetes-induced upregulation of UCP- 2 results in excessive uncoupling and reduced oxidative stress in the kidney via activation of ANT.

## Introduction

The prevalence of diabetic nephropathy is increasing rapidly world-wide [1], but presently there is no treatment and approximately 45% of all cases of end-stage renal disease are due to diabetic nephropathy [2]. The recent focus of mechanisms underlying diabetic nephropathy has shifted from the glomerulus to the proximal tubule [3]. The kidney proximal tubule performs a majority of the active transport in the kidney, which requires a high ATP production and a high cellular content of mitochondria. The ensuing high rate of oxidative phosphorylation is a potential source of superoxide radicals since an estimated 0.1–0.2% of the mitochondrial O_2_ usage results in superoxide formation. Increased passage of electrons down the respiratory chain increases the mitochondria membrane potential and therefore also formation of superoxide [4].

Mitochondria uncoupling may be a protective mechanism to counter increased mitochondria superoxide formation. Shunting of protons across the inner mitochondrial membrane lowers the membrane potential and limits superoxide formation. However, O_2_ consumption required for proton transport uncoupled from ATP production will be added to that required for oxidative phosphorylation and therefore increases total O_2_ consumption. The level of mitochondria uncoupling can be evaluated in isolated mitochondria during ATP-synthase inhibition [5]. Then, the addition of electron-donating substrates such as glutamate can increase O_2_ consumption only if an uncoupling mechanism is present. This is denoted as glutamate-stimulated O_2_ consumption of isolated mitochondria in the present study. There are five different isoforms of uncoupling proteins (UCP) known to mediate mitochondria uncoupling [6], but UCP-2 is the isoform expressed in both rat and human kidneys [5], [7] where it is reported to mediate mitochondria uncoupling in the diabetic kidney [5]. Whereas reduced superoxide formation, via mitochondria uncoupling, may protect the diabetic kidney from damaging oxidative stress, the concomitantly increased O_2_ consumption may result in hypoxia and contribute to the development of diabetic nephropathy. Indeed, kidney tissue hypoxia in diabetes has been reported [8]. The present study investigates the role of UCP-2 in the regulation of mitochondria function and oxidative stress in the diabetic kidney by applying *in vivo* siRNA-mediated knockdown of UCP-2.

## Results

**Figure 1:**
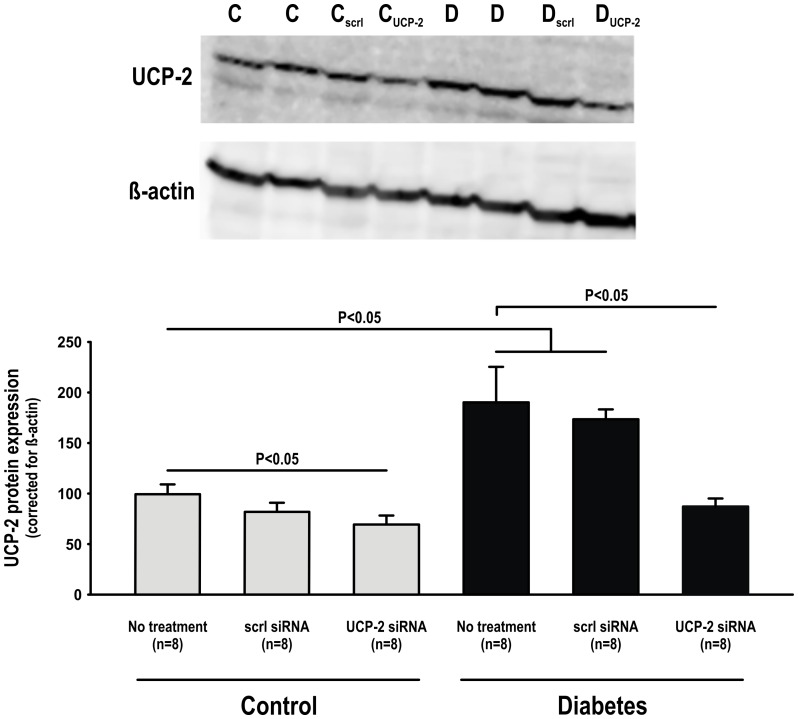
UCP-2 protein expression in control and diabetic animals with and without scramble or UCP-2 siRNA administration. A representative blot is displayed on the top.

**Figure 2:**
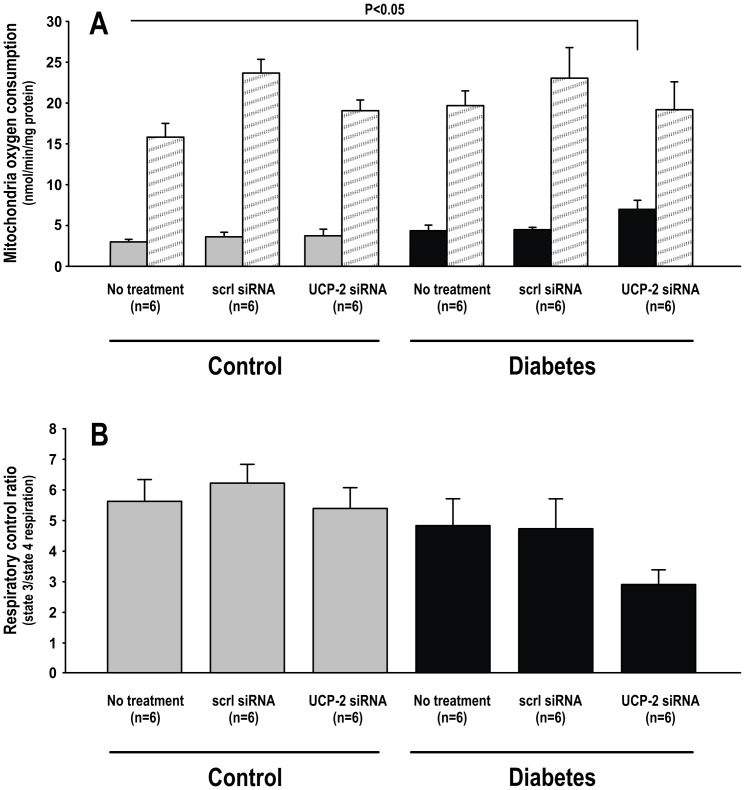
Mitochondria oxygen consumption measurements A) during state 4 (filled bars) and state 3 (patterned bars) in control and diabetic animals with and without scrambled or UCP-2 siRNA. B) Respiratory control ratio (state 3/state 4 respiration) in control and diabetic animals with and without scrambled or UCP-2 siRNA.

UCP-2 protein expression was increased in the kidneys of diabetic rats but siRNA resulted in −30% decreased expression compared to baseline in control animals and −55% compared to baseline in diabetic animals. Scrambled siRNA did not significantly alter UCP-2 protein expression in any of the groups compared to corresponding untreated animals (Fig. 1). Diabetic animals displayed increased blood glucose levels compared to control animals. siRNA did not affect either blood glucose levels or body weights (Table 1). Diabetic animals administered UCP-2 siRNA displayed increased state 4 respiration compared to untreated controls, whereas scramble siRNA had no effect. State 3 respiration and RCR of isolated mitochondria did not differ between any of the groups (Fig. 2).

**Table 1:** Blood glucose and body weight in control and diabetic animals with and without siRNA administration.

	Body weight (g)	Blood glucose (mmol/l)
Control	349±7	4.8±0.1
Control+scrl siRNA	322±12	4.9±0.4
Control+UCP-2 siRNA	304±7	5.7±0.5
Diabetes	278±8	20.1±1.2*
Diabetes+scrl siRNA	296±9	20.9±1.2*
Diabetes+UCP-2 siRNA	307±13	22.9±1.1*

* denotes p<0.05 vs. untreated control animals.

**Figure 3:**
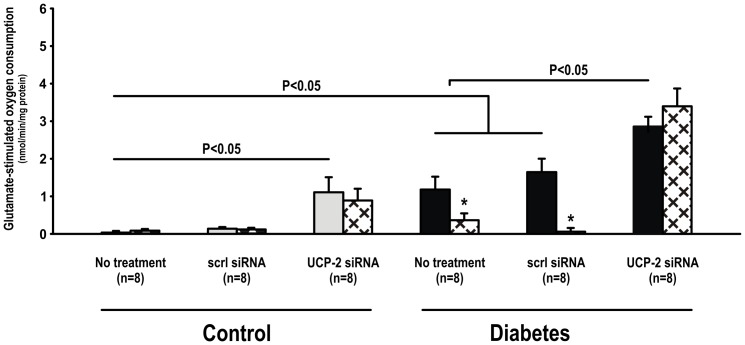
Mitochondria uncoupling measured as glutamate-stimulated O_2_ consumption during inhibition of the ATP-synthase with oligomycin during baseline (filled bars) and after inhibition of UCP-2 with GDP (patterned bars) in control and diabetic animals with and without scrambled or UCP-2 siRNA. * denotes p<0.05 vs. baseline within the group.

**Figure 4:**
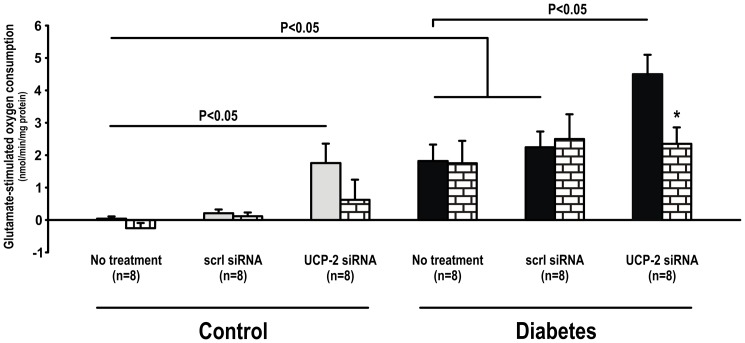
Mitochondria uncoupling measured as glutamate-stimulated O_2_ consumption during inhibition of the ATP-synthase with oligomycin during baseline (filled bars) and after inhibition of ANT with ADP (patterned bars) in control and diabetic animals with and without scrambled or UCP-2 siRNA. * denotes p<0.05 vs. baseline within the group.

**Figure 5:**
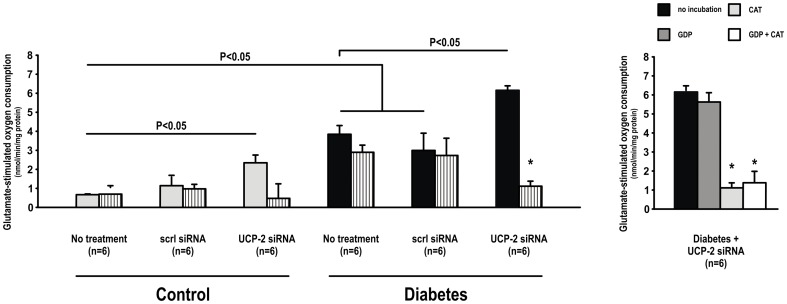
Mitochondria uncoupling measured as glutamate-stimulated O_2_ consumption during inhibition of the ATP-synthase with oligomycin during baseline (filled bars) and after inhibition of ANT with CAT (patterned bars) in control and diabetic animals with and without scrambled or UCP-2 siRNA. * denotes p<0.05 vs. baseline within the group.

Mitochondria glutamate-stimulated O_2_ consumption was increased in mitochondria isolated from the kidneys of untreated diabetic rats compared to corresponding controls. UCP-2 siRNA, but not scrambled siRNA, increased glutamate-stimulated O_2_ consumption in both controls and diabetics. GDP inhibited glutamate-stimulated O_2_ consumption in untreated diabetics and diabetic animals receiving scrambled siRNA. No effect of GDP was observed in any of the control groups (Fig. 3). ADP inhibited glutamate-stimulated O_2_ consumption in mitochondria isolated from diabetic animals treated with UCP-2 siRNA, but had no effect in any of the other groups (Fig. 4). CAT inhibited glutamate-stimulated O_2_ consumption in both control and diabetic animals treated with UCP-2 siRNA, but did not affect glutamate-stimulated O_2_ consumption in any of the untreated animals or animals treated with scrambled siRNA (Fig. 5).

**Figure 6:**
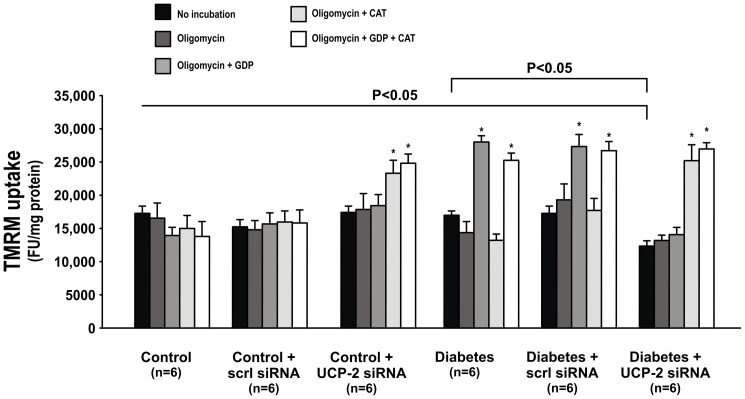
Mitochondria membrane potential measured as TMRM-uptake before and after GDP, CAT or the combination of CAT and GDP during baseline and inhibition of the ATP-synthase in control and diabetic animals with and without scrambled or UCP-2 siRNA. * denotes p<0.05 vs. no incubation within the group.

Baseline TMRM-uptake in presence of oligomycin was similar in all control groups and untreated diabetics. Baseline TMRM-uptake was lower in diabetic animals treated with UCP-2 siRNA compared to all other groups. GDP increased TMRM-uptake in untreated diabetic animals, but had no effect in any of the other groups. Furthermore, CAT increased TMRM-uptake in UCP-2 siRNA-treated diabetic animals whereas CAT had no effect in any of the other groups (Fig. 6).

**Figure 7:**
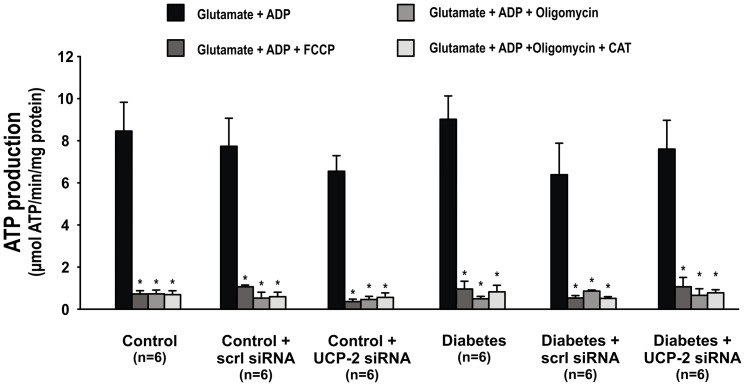
Mitochondria ATP-production during no incubation and after oligomycin, FCCP or CAT in control and diabetic animals without scrambled or UCP-2 siRNA. * denotes p<0.05 vs. no incubation within the group.

Mitochondria ATP production was similar in all groups, and substantially lower in the presence of either FCCP, oligomycin or the combination of oligomycin and CAT (Fig. 7).

**Figure 8:**
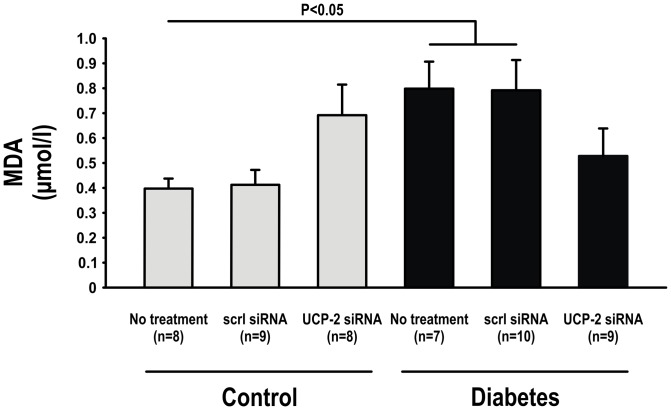
Whole-body oxidative stress levels measured as circulating MDA levels in control and diabetic animals with and without scrambled or UCP-2 siRNA.

**Figure 9:**
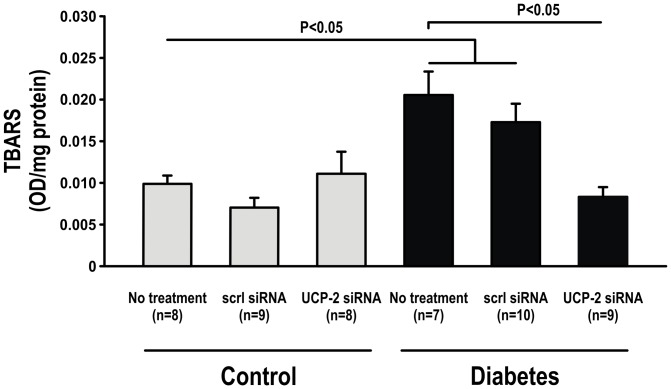
Kidney tissue oxidative stress levels measured as TBARS in control and diabetic animals with and without scrambled or UCP-2 siRNA.

Plasma MDA levels were elevated in untreated diabetic animals compared to controls but unaffected by UCP-2 siRNA (Fig. 8). TBARS in kidney cortex was increased in untreated diabetics, but decreased in the diabetic animals administered UCP-2 siRNA. Neither UCP-2 siRNA to controls nor scrambled siRNA to any of the groups altered kidney tissue TBARS (Fig. 9).

## Discussion

The main new finding from the present study was that mitochondria uncoupling directly can regulate mitochondria membrane potential and oxidative stress in the diabetic kidney.

Baseline mitochondria uncoupling in the diabetic kidney was evident as increased UCP-2 expression, glutamate-stimulated O_2_ consumption and increased mitochondria membrane potential after UCP-2 blockade by GDP, which is consistent with our previous report [5]. However, UCP-2 knockdown in diabetic animals resulted in a paradoxical increase in uncoupling evidenced by increased glutamate-stimulated O_2_ consumption. This was a distinct uncoupling pathway since it was unaffected by blockade of UCP-2 with GDP. Since this increased uncoupling after siRNA to UCP-2 was inhibited by blockade of the ANT with ADP or CAT, we concluded that it was mediated via the ANT. The failure of GDP to inhibit this uncoupling excludes a role for residual UCP-2. During normal function, ANT exchanges ADP for ATP across the mitochondria inner membrane [9] and alters between two conformations (c and m-conformation) as translocation occurs. However, ANT can mediate mitochondria uncoupling under certain conditions. The amount of mitochondrial ANT has been reported to determine the level of basal uncoupling [10] and uncoupling via the ANT can be induced by fatty acids [11].

It was surprising that the elevated mitochondria uncoupling in UCP-2 siRNA-treated diabetics was inhibited by addition of ADP after blocked ATP-synthesis since, in absence of ATP, the ANT pathway should be inactive. However, ATP also is produced by the citric cycle. Indeed, although mitochondria ATP production during ATP-synthase inhibition was substantially reduced, some ATP production remained. Presumably, this was sufficient to exchange for ADP and induce ANT-mediated mitochondrial uncoupling. However, the ANT may lock in c-conformation in the presence of oligomycin and excess ADP or CAT. This would inhibit proton leakage and reduce mitochondria uncoupling. Moreover, in potato tuber mitochondria the ANT can import double stranded DNA independently of ATP-synthesis i.e. unaffected by oligomycin alone. Importantly, locking the ANT in c-conformation with either oligomycin and ADP or CAT significantly reduced DNA import [12]. In mitochondria isolated from siRNA-treated diabetic rats the ANT provides a second ATP-independent uncoupling pathway, presumably to regulate oxidative stress. Locking the ANT in c-conformation had no effects in untreated diabetic animals, demonstrating that ANT is not causing the mitochondrial uncoupling in these animals.

Mitochondria uncoupling was unaffected by ADP in untreated diabetic rats even though ADP has been reported to inhibit UCP [13]. However, the lack of UCP-2 inhibition by ADP has previously been reported in kidney mitochondria from diabetic rats [5] and, to the best of our knowledge, inhibition of UCP-2-mediated uncoupling by ADP has only been studied during normal conditions. Therefore, future studies are needed in order to evaluate if ADP actually can inhibit UCP-2 function in diabetic kidney mitochondria.

UCP-2 knockdown in control animals resulted in increased mitochondria uncoupling, which paradoxically did not result in decreased mitochondrial membrane potential. It may be speculated that glutamate (i.e. electron-donating NADH) is present in sufficient amount to counteract relatively small changes in uncoupling activity and therefore maintain normal membrane potential. This speculation is supported by the results showing increased TMRM uptake after addition of CAT to these mitochondria.

Mitochondria membrane potential, as indicated by TMRM-uptake, was not increased in untreated diabetic animals, which is contrary to previous reports [14], [15]. These studies were performed on isolated mitochondria under conditions reflecting those of control animals and therefore do not reflect the *in vivo* situation of hyperglycemia and increased mitochondria substrate load. Incubation with GDP increased membrane potential in untreated diabetics, directly demonstrating a regulatory role of mitochondria uncoupling via UCP-2 in the regulation of mitochondria membrane potential. Interestingly, increased ANT-mediated uncoupling in the UCP-2 siRNA-treated diabetics resulted in significantly lower mitochondrial membrane potential. Importantly, CAT increased mitochondria membrane potential in diabetic animals administered UCP-2 siRNA, which further supports a crucial compensatory involvement of ANT after acutely reduced UCP-2 function.

Increased mitochondria uncoupling may be a protective mechanism to regulate mitochondria superoxide formation and prevent excessive oxidative damage in the diabetic kidney [16]. Indeed, the increased ANT-mediated uncoupling after UCP-2 knockdown reduced oxidative stress in the diabetic kidney. However, this may to be a kidney specific mechanism since although whole-body oxidative stress, indicated by circulating MDA-levels was increased in diabetes, it was not significantly reduced by siRNA to UCP-2. The explanation for the discrepancy between kidney and whole-body regulation of oxidative stress in diabetes may relate to specific role of UCP-2 in the kidney. However, UCP-2 is the only isoform in the kidney whereas in other tissues UCP-3 and other isoforms will presumably not be affected by UCP-2 siRNA. Therefore, compensatory uncoupling via ANT in other tissues body may not occur to the same extent as much in other tissues, resulting in less effect on circulating markers of oxidative stress. Also, reactive oxygen species are produced by several different sources and the kidney proximal tubule is especially influenced by mitochondria-derived superoxide formation due to its high metabolic demand. It is therefore possible that the beneficial effect of mitochondria uncoupling in reducing oxidative stress is more pronounced in the kidney compared to the whole body. It seems likely that even though oxidative stress levels are increased in diabetes mitochondria uncoupling may present a mechanism to limit an even more exaggerated oxidative stress level in the diabetic kidney.

The results from the present study confirm the importance of mitochondria uncoupling in regulating mitochondrial oxidative stress. Indeed, when uncoupling via UCP-2 was inhibited the uncoupling function was rapidly and quantitatively replaced by a nascent uncoupling via ANT. A similar compensatory mitochondria uncoupling via ANT has been reported from skeletal muscle mitochondria of UCP-3 knockout-mice. Two weeks caloric restriction resulted in increased CAT-sensitive uncoupling and it was proposed that increased ANT uncoupling compensates for the absence of UCP-3 [17]. Furthermore, ANT is expressed in the rat kidney [18] and is activated by fatty acids, alkenals such as 4-hydroxynonenal [19] and AMP [20]. Oxidative stress in diabetes increases lipid peroxidation and an enhanced free fatty acid production would be expected to increase ANT activity. Thus, increased ANT activation via lipid peroxidation products, may explain the increased kidney mitochondria uncoupling after UCP-2 siRNA. We speculate that ANT compensates for decreased UCP-2 function to protect the mitochondria from excessive diabetes-induced oxidative stress. Indeed, Duval *et al.* demonstrated that UCP-2 siRNA increased mitochondria superoxide production in murine endothelial cells [21]. It is possible that the initial event after UCP-2 siRNA administration in our study is increased mitochondrial superoxide production, leading to lipid peroxidation and activation of ANT-mediated mitochondria uncoupling. Remarkably, the compensatory ANT-mediated uncoupling is more potent compared to the UCP-2-mediated uncoupling in untreated diabetic animals, as evident from both the lower mitochondrial membrane potential and the reduced oxidative stress in the kidney of these animals. However, increased mitochondria uncoupling results in increased mitochondrial O_2_ consumption. It should be noted that the normal kidney O_2_ consumption is high already during baseline conditions due to the high metabolic demand for active tubular transport. This will be further elevated in diabetes due to enhanced proximal transport without a corresponding increase in blood flow [16], resulting in tissue hypoxia throughout the diabetic kidney [8]. Kidney hypoxia is strongly implicated as a common pathway for end-stage renal disease [22] and it may be speculated that although a further increased mitochondria uncoupling via the ANT results in lower oxidative stress, the concomitantly increased O_2_ consumption will accelerate kidney tissue hypoxia and development of diabetic nephropathy.

**Figure 10:**
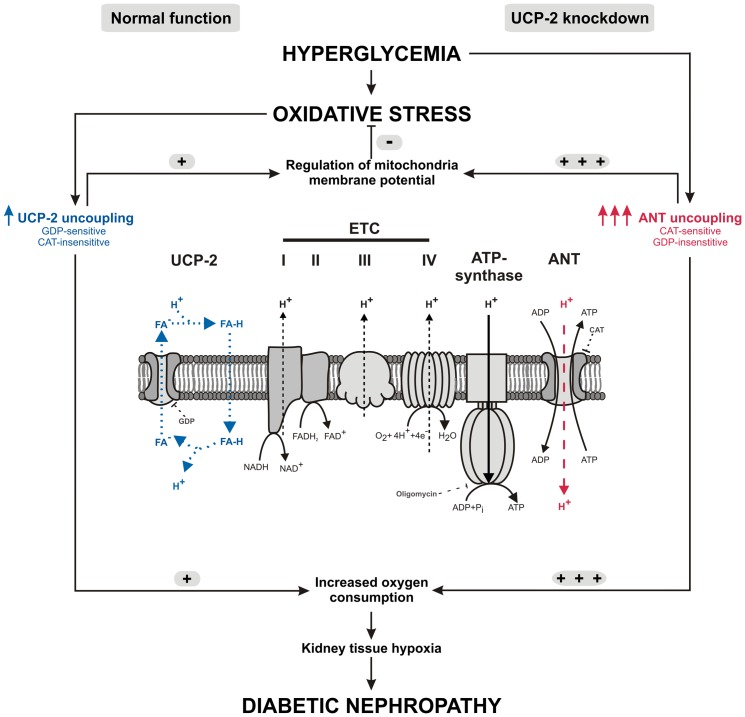
Summary of proposed mechanisms. Pathways highlighted in blue denote uncoupling via UCP-2; pathways highlighted in red denote uncoupling via ANT. ANT – adenosine nucleotide transporter, CAT – carboxyatractylate, ETC – electron transport chain, FA^−^ – charged fatty acid, FA-H – uncharged fatty acid, GDP – guanosine diphosphate, UCP-2 – uncoupling protein 2.

In conclusion, the present study demonstrates that UCP-2 is directly involved in regulating mitochondrial membrane potential and therefore also is a potential mechanism to prevent excessive mitochondria superoxide formation in the diabetic kidney. Abolished UCP-2-mediated mitochondria uncoupling results in an even more potent ANT-mediated uncoupling which reduces membrane potential and oxidative stress damage in the diabetic kidney. However, mitochondria uncoupling increases total O_2_ consumption and can potentially amplify the already existing kidney tissue hypoxia in diabetes and therefore accelerate development of diabetic nephropathy (Fig. 10).

## Materials and Methods

### Animal procedures

All animal procedures were carried out in accordance with the recommendations in the Guide for the Care and Use of Laboratory Animals of the National Institutes of Health. The protocol was approved by the Georgetown University Animal Care and Use Committee (protocol number 07-125) and all efforts were made to minimize suffering. All animals had free access to standard rat chow and tap water. Diabetes was induced in male Sprague Dawley rats (250–300 g) by intravenous injection of streptozotocin (65 mg/kg bw dissolved in 0.2 ml saline) into the tail vein. Blood glucose was measured using a reagent test strip (MediSense, Bedford, MA, USA) in a blood sample obtained from the cut tip of the tail and the animals were considered diabetic if blood glucose levels increased ≥15 mM within 24 h of streptozotocin administration, and remained elevated.

Under isoflouran anaesthesia (2% in 40% O_2_) a polyethylene catheter was inserted into in the carotid artery and a non-functional scrambled siRNA or siRNA targeting UCP-2 (100 µg/rat; id nr 50931, Ambion, Austin, TX, USA, Table 2) was administered in a total volume of 6 ml 37°C sterile saline during 6 seconds. The carotid artery was ligated and the wound closed. siRNA was administered at day five of diabetes and all experiments carried out two days thereafter.

**Table 2:** Sequence of siRNA towards UCP-2 and scrambled siRNA.

siRNA target	Sequence (5′- 3′)
Scramble (no known target)	AAGCTTCATAAGGCGCATAGC
UCP-2	GGAGAGAGUCAAGGGUCAGtt

### Verification of successful UCP-2 knockdown using western blot

Kidney cortex were homogenized in RIPA buffer (1% tergitol type NP40, 0.5% sodium deoxycholate, 0.1% SDS, 10 mmol/l NaF, 80 mmol/l Tris, pH 7.5) with enzyme inhibitors (Phosphatase inhibitor cocktail-2; 10 µl/ml, Sigma-Aldrich, St Louis, MO, USA and Complete Mini; 1 tablet/1.5 ml; Roche Diagnostics, Mannheim, Germany) added fresh before each experiment. Molecular weight separation was performed on 12.5% Tris-HCl gels with Tris/glycine/SDS buffer, the proteins transferred to nitrocellulose membranes and UCP-2 detected with goat anti-rat UCP-2 (1∶1000; Santa Cruz Biotechnology, Santa Cruz, CA, USA) and HRP-conjugated rabbit anti-goat (1∶10,000; Kirkegaard and Perry Laboratories, Gaithersburg, MD, USA). Luminescent signal was captured on an ECL-camera system (Kodak image station 2000; New Haven, CT). β-actin was detected with mouse anti-rat β-actin antibody (1∶10,000, Sigma-Aldrich, St Louis, MO, USA) and secondary HRP-conjugated goat-anti mouse antibody (1∶60,000; Kirkegaard and Perry Laboratories, Gaithersburg, MD, USA).

### Mitochondria isolation and functional assessment

Under isoflourane anaesthesia (2% in 40% O_2_), a blood sample was collected and frozen for later analysis of plasma malondialdehyde (MDA) and the kidneys excised and rapidly placed in ice-cold buffer A (in mmol/l: 250 sucrose, 1 EGTA, 10 4-(2-hydroxyethyl)-1-piperazineethanesulfonic acid (HEPES), pH 7.4, 300 mOsm/kg H_2_O). Isolation of kidney mitochondria were performed as previously described [5]. In brief, kidney cortex was dissected out on ice, rinsed with buffer A and homogenized in 10 ml of buffer A on ice using a Potter-Elvehjem homogenizer (800 rpm). The homogenate was transferred to pre-chilled centrifuge-tubes, centrifuged at 800×g for 10 min at 4°C. The supernatants were transferred to new tubes and centrifuged at 8000×g for 10 min at 4°C. The pellets were gently resuspended in buffer A now containing 1 mg/ml of bovine serum albumine (BSA, further purified faction V, fatty acid free) and centrifuged at 8000×g for 10 min at 4°C. The final pellet was dissolved in experimental buffer (in mmol/l: 220 mannitol, 70 sucrose, 5 MgCl_2_, 5 KPO_4_^−^, 10 HEPES, 0.048 sodium palmitate, 1 mg/ml fatty acid free BSA, pH 7.4 and 330 mOsm/kg H_2_O).

Mitochondria O_2_ consumption was measured using a custom-made gastight plexi-glass chamber with a total volume of 1.100 ml thermostatically controlled to 37°C. The chamber was continuously stirred with an air-driven magnetic stirrer. A modified Unisense 500 O_2_ sensing electrode (Unisense, Aarhus, Denmark) was calibrated with air-equilibrated buffer solution set to 228 µmol/l O_2_, Na_2_S_2_O_5_-saturated buffer set to zero and rate of O_2_ disappearance recorded. At the end of each experiment, a sample was taken to determine the protein concentration using DC Protein Assay (Bio-Rad Laboratories, Hercules, CA, USA). O_2_ consumption was calculated as the disappearance rate of O_2_ adjusted for protein concentration.

Addition of glutamate (10 mmol/l) and adenosine diphosphate (ADP, 400 µmol/l) and subsequently calculated respiratory control ratio (RCR; O_2_ consumption after ADP divided by O_2_ consumption after glutamate) was used as indication of functional mitochondria. Any increased O_2_ consumption after addition of glutamate to mitochondria pre-incubated with the ATP-synthase inhibitor oligomycin (12 µg/mg protein) indicates O_2_ consumption unrelated to ATP production and was used as an indicator of mitochondrial uncoupling. This is referred to as glutamate-stimulated O_2_ consumption. In separate experiments, ADP (400 µmol/l), guanosine diphosphate (GDP; inhibitor of UCP [13], [23], 500 µmol/l) or CAT (inhibitor of ANT [10], 0.5 µmol/l) was added in the presence of oligomycin and glutamate-stimulated O_2_ consumption analysed. In experiments using CAT, the mitochondria O_2_ consumption was recorded using the Oroboros Oxygraph-2k (Oroboros Instruments, Innsbruck, Austria).

### Mitochondria membrane potential

Mitochondria membrane potential was measured as uptake of the flourofor tetramethylrhodamine methyl ester (TMRM) [24]. TMRM (0.35 µmol/l) was mixed with mitochondria experimental buffer and fluorescence measured at excitation 546 nm and emission 590 nm in a 384-well plate (GreinerBio One, Frickenhausen, Germany), and denoted total TMRM (TMRM_T_). Mitochondria incubated with oligomycin and glutamate or with coincubation of oligomycin, glutamate, GDP and CAT were added to the wells, incubated for 5 min and pelleted at 8000×g for 10 min at room temperature. The supernatant of each pellet was analyzed for fluorescence (TMRM outside; TMRM_O_). Mitochondria uptake of TMRM was calculated as TMRM_T_-TMRM_O_ and corrected for protein concentration.

### ATP production by isolated mitochondria

Mitochondria ATP production was analyzed with a commercially available bioluminescence assay from Molecular Probes (ATP determination kit, A22066, Molecular Probes, Invitrogen, Paisley, UK) according to manufacturer's instruction. Analysis was performed in four different settings: 1) mitochondria, glutamate and ADP, 2) mitochondria, glutamate, ADP and carbonylcyanide-p-trifluoromethoxyphenyl-hydrazone (FCCP), 3) mitochondria, glutamate, ADP and oligomycin, and 4) mitochondria, glutamate, ADP, oligomycin and CAT. Samples were incubated for 3 min at 37°C and thereafter snap frozen in liquid nitrogen. ATP production was corrected for protein concentration and expressed as µmol ATP/min/mg protein.

### Thiobarbituric Acid Reactive Substances (TBARS) in kidney cortex homogenate

TBARS was measured as previously describe [8]. In brief, 50 µl cortex homogenate was added to 500 µl hydrochloric acid (50 mmol/l), vortexed and 167 µl thiobarbituric acid (0.67%) added. After incubation for 30 min at 95°C the samples were cooled to room temperature and 667 µl methanol∶n-butanol added (3∶17 mix, prepared fresh). The sample vortexed and centrifuged at 2500 rpm for 20 min at 18°C. The top layer was transferred to a transparent 384 well plate, analyzed for absorbance at 535 nm and corrected for protein concentration.

### Malondialdehyde (MDA) in plasma

Free plasma MDA was measured by HPCE-Micellar Electrokinetic Chromatography. Briefly, plasma was filtered through a centrifugal filter with 3 kD cut-off. The ultrafiltrate was directly injected into an uncoated fused silica capillary (75 micron ID, length to detector 40 cm, total length 50.2 cm) on a Beckman Coulter MDQ system (Fullerton, CA, USA) equipped with a UV detector. A large volume stacking was used to introduce a large plug of the sample hydrodynamically (0.5 psi, 20 seconds). The background electrolyte solution contained sodium tetraborate (25 mmol/l), spermine HCl (1 mmol/l), and tetradecyltrimethylammonium bromide (TTAB; 2 mmol/l) at a pH of 9.7. UV detection was carried out at 260 nm and Methyl MDA was used as an internal standard. The separation was carried out at −12 kV and 25°C. Intra-assay and inter-assay CV for this assay in samples of plasma are 2.1% and 4.3% respectively and the limit of detection is 0.1 µmol/l.

### Statistical analysis

Statistical comparisons were made using one-way analysis of variance (ANOVA) followed by Bonferroni's multiple comparisons test. Paired Student's t-tests and repeated one-way ANOVA were applied for comparisons within each group. A p<0.05 was considered statistically significant and all values are presented as mean±SEM.
